# Process evaluation of specialist nurse implementation of a soft opt-out organ donation system in Wales

**DOI:** 10.1186/s12913-019-4266-z

**Published:** 2019-06-24

**Authors:** Jane Noyes, Leah Mclaughlin, Karen Morgan, Abigail Roberts, Bethan Moss, Michael Stephens, Phillip Walton

**Affiliations:** 1 School of Social Sciences, Bangor University, Room 107, Neuaddc Ogwen, Bangor, Wales, UK LL57 2DG; 2 Major Health Conditions Policy Team, Directorate of Health Policy, Health and Social Services Group, Welsh Government, Cardiff, Wales, UK CF10 3NQ; 3 NHS Blood and Transplant, North West Regional Office, 14 Estuary Banks, Speke, Liverpool, L24 8RB UK; 4 NHS Blood and Transplant, South Wales, South West and South Central, Unit 3 Cae Gwyrdd, Greenmeadow Springs Business Park, Tongwynlais, Cardiff, CF15 7AB UK; 5 Department of Nephrology and Transplantation, Heath Park, Cardiff and Vale University Health Board, University Hospital of Wales, Cardiff, CF14 4XW UK

**Keywords:** Organ donation, Soft opt-out, Consent, Nursing, Process evaluation, Qualitative, Wales

## Abstract

**Background:**

Wales introduced a soft opt-out organ donation system on 1st December 2015 with the aim of improving consent rates. In the first 18 months consent rates improved but the difference could not solely be attributed to the soft opt-out system when compared with similar improvements in consent rates in other UK nations.

****Methods**:**

We conducted an 18 month post-intervention qualitative process evaluation involving 88 family members of 60/211 potential organ donor cases, and 19 professionals. Views and experiences of Specialist Nurses in Organ Donation who implemented the new system and family members who were involved in decision making were collected to see how their respective behaviours impacted on implementation. Data collection included interviews, focus groups and qualitative questionnaire data.

****Results**:**

Implementation was considered a success by Specialist Nurses in Organ Donation. The bespoke retraining programme and responsive approach to addressing initial implementation issues were identified as examples of best practice. Specialist Nurses in Organ Donation were valued by family members. Six implementation issues had an impact on consent rates – the media campaign had gaps, the system was more complex, challenges in changing professional behaviours, inability to obtain the required standard of evidence from family members to overturn a donation decision, increased complexity of consent processes, and additional health systems issues.

****Conclusion**:**

This is the first comprehensive process evaluation of implementing a soft opt-out system of organ donation. Specific elements of good implementation practice (such as investment in the retraining programme and the responsiveness of Specialist Nurses in Organ Donation and managers to feedback) were identified. The key message is that despite retraining, nursing practice did not radically change overnight to accommodate the new soft opt-out system. Policy makers and health service managers should not assume that nurses simply need more time to implement the soft-out as intended. Additional responsive modification of processes, ongoing training and support is required to help with implementation as originally intended. Scotland, England and the Netherlands are introducing soft opt-out systems. There is an opportunity to learn from initial implementation in Wales, by acknowledging gaps, good practice and opportunities to further improve processes and nursing practices.

**Electronic supplementary material:**

The online version of this article (10.1186/s12913-019-4266-z) contains supplementary material, which is available to authorized users.

## Background

Increasing the number of organs for transplant is a global clinical and research priority [1]. One strategy adopted by many governments has been to move to an ‘opt-out’ system (i.e. presumed consent to organ donation, unless a person actively opts out) from an ‘opt-in system’ (i.e., the default is to be a non-donor unless a person actively opts in) [2]. There are two types of opt-out system: a ‘hard’ opt-out where the family are not consulted or a ‘soft’ opt-out where the family are consulted and have the final say. Soft-opt out systems vary in the way they are designed and implemented and how they are intended to work [3]. The move to an opt-out system has been justified as a way of changing population behaviour along with evidence that countries with opt-out systems tend to have higher transplantation rates than those with opt-in systems [4].

The role of nurses in organ donation is conceptualised and organised differently across countries. In the United Kingdom (UK), National Health Service Blood and Transplant (NHSBT) is a Special Health Authority that is responsible for blood, tissue, organ donation and transplantation services. Specialist Nurses in Organ Donation (SNODs) are employed and trained by NHSBT. The role was conceived as a senior nurse post and SNODs would normally be recruited from critical care roles in NHS hospitals [5]. The role is outlined in Table 1.

**Table 1:** The main responsibilities of the specialist nurse in organ donation at the time of the study. Adapted from [6]

Main responsibilities of the Specialist Nurse in Organ Donation at the time of the study				
Consent related activity	Clinical activity	Theatre	Hospital development	Potential donor audits
Triage incoming referrals Attend the referrals Approach families for consent	Engage with all clinical activity following consent Provide support for family members and staff	Attend theatre and help coordinate the retrieval procedure	Engage with hospitals to drive referrals to ensure hospitals comply with transplant process Engage in education and practice development activities	Audit files of all people who die in the Emergency Department and Intensive Care Unit below the age of 81

The hospital triggers a referral to NHSBT when a patient is identified as a potential organ donor. NHSBT assess the referral and if appropriate mobilise a SNOD. In the UK, the initial conversation about organ donation (the ‘approach’) is undertaken by key professionals from the patient’s multi-disciplinary team. This should include at minimum the SNOD and the consultant. The bed side nurse or other clinical staff may also be involved depending on the circumstances. Typically, the consultant will begin the ‘breaking bad news’ conversation. Thereafter the SNOD is expected to be a key member of the multi-disciplinary team who liaises with the family member(s) in any conversations about organ donation [7]. The circumstances of a death where donation is a possibility are often traumatic (e.g. unexpected and sudden brain injury). When organ donation is an option, family members are brought together in rare and unique circumstances. The SNOD role has a unique emphasis on taking and establishing legal consent and completing the associated documentation. Evidence suggests that SNOD intervention with traumatised families can help increase the organ donation consent rate [7].

### Implementing the opt-out system in Wales

Wales has a population of just over 3 million people and a devolved parliamentary legislature within the UK, that implemented a soft opt-out system on 1st December 2015 [8, 9]. Under the new soft opt-out system every person who meet specific criteria are considered potential organ donors.

Implementing the new soft opt-out system (the intervention) had three components: the Act; public media campaign, and retraining specialist nurses and other key members of NHS staff. A detailed description of the components and how the intervention is intended to work can be found in the study protocol [10]. In brief, the programme theory was that these three components would work together to support family member(s) to put aside their own views and support the organ donation decision made by the deceased person during their lifetime. Welsh citizens were encouraged via a media campaign to make their decision known by; registering it on the Organ Donor Register (ODR) or, verbally expressing it to family member(s) or, doing nothing and consent would be presumed (deemed consent). People could also appoint a representative to convey their donation decision for them. The key intended changes were that unless a person opted out they were presumed to be an organ donor, and family member(s) were no longer the decision makers for deceased potential organ donors in Wales. SNODs were expected to support family member(s) to uphold the deceased person’s decision that was made during life. A comparison of the old and new system is outlined in Table 2.

**Table 2:** Comparison of previous opt-in and new soft out-opt system. Adapted from [11]

Decision Type					
	Active	Passive	Family consent	Geographical reach	Role of family
Former Opt-in system	Register to opt in on the organ donor register Verbally tell a relative or friend you want or do not want to be a donor Write telling a relative or friend you want or do not want to be a donor Nominate a representative to make the decision for you. (Nowhere to record this decision)	Do nothing and remain a non-donor unless your relative gives consent to organ donation.	Person under 18, lacks mental capacity	UK wide	To give assent or agreement or veto for organ donation if their relative has actively opted in, or to make a donation decision on behalf of their relative.
New Opt-Out System in Wales	Register to opt-in on the organ donor register Verbally tell a relative or friend you want to be a donor Write telling a relative or friend you want or do not want to be a donor Register to opt-out on the organ donor register Appoint a patient representative on the organ donor register to make the decision for you	Do nothing and remain as a donor (Deemed consent)	Person under 18, lacks mental capacity	Wales only Welsh citizens have to die in Wales for the soft-opt out to apply. If they die in England the opt-in system applies.	To support the donation decision of their relative made in life. Clinicians acknowledged that they would not pursue organ donation if the family member refused to support their relative’s donation decision made in life as the family still have a legal right to override their relative’s decision.

The SNOD role was empowered though the Human Tissue Authority with putting the new law into action by implementing the soft opt-out system in Wales. The associated Code of Practice placed responsibility on SNODs for ensuring legal consent in the new soft-opt out system in Wales. Of all the professionals involved in implementing the new soft opt-out system, SNODs had most pressure placed upon them to increase organ donation. Therefore a process evaluation of specialist nurse implementation of a soft opt-out organ donation system is essential.

### Initial outcomes

**Fig. 1:**
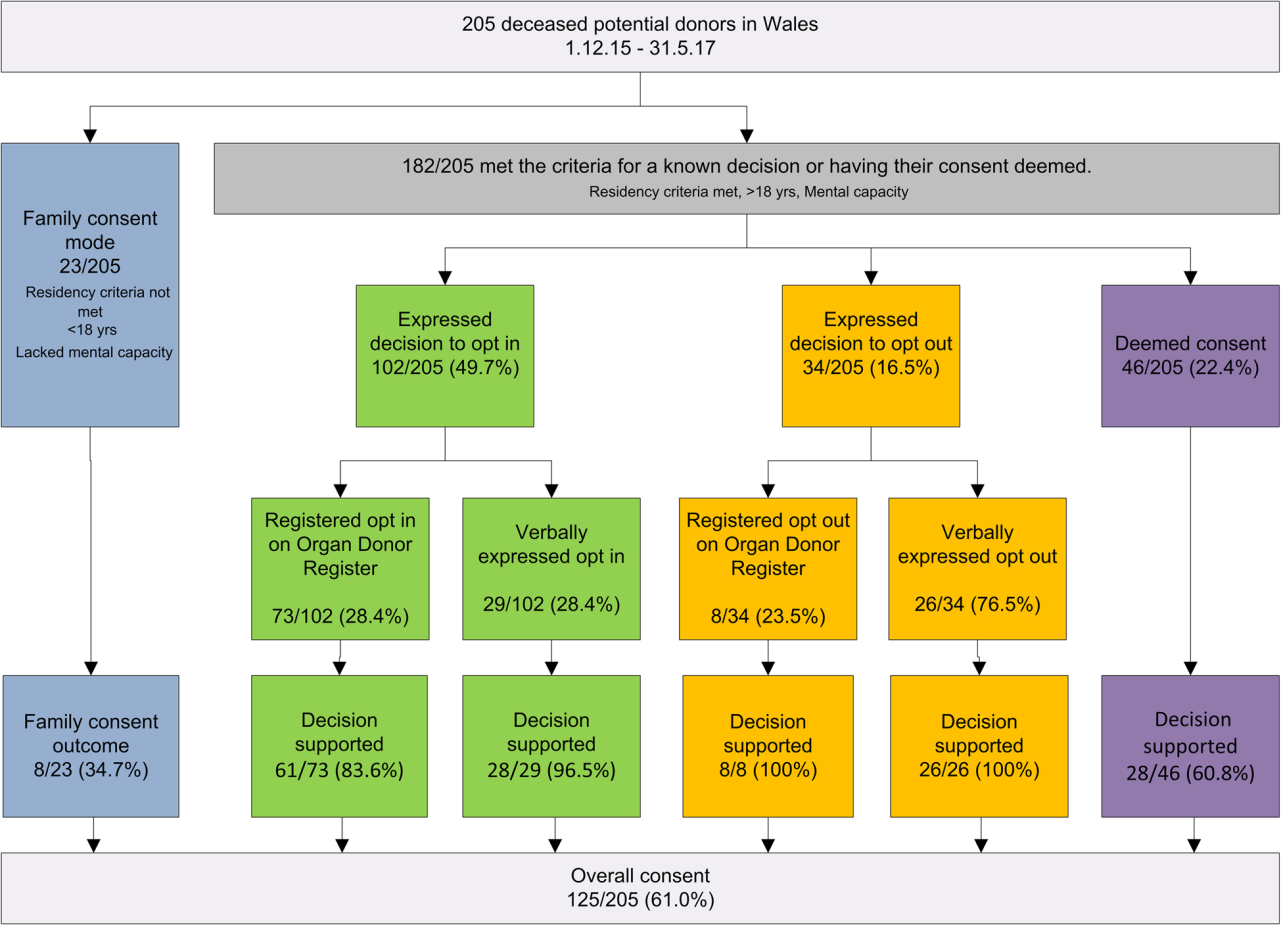
Consent outcomes for 205 potential organ donors in Wales. Shows a consort-type diagram of consent decisions. Reproduced from [11]

In order to contextualise the process evaluation findings, we reproduce below a summary of the initial outcomes from the before and after study [11]. All 205 potential organ donor cases in Wales were tracked from 1st December 2015 for 18 months [11]. 182/205 cases met the criteria for a known decision (registered or verbally expressed decision made during life) or having their consent deemed. In addition, 6/38 potential Welsh organ donor cases who died in English hospitals were purposively sampled and followed up (making 211 cases in total). Using routinely collected bespoke data, cases were grouped using descriptive statistics (total number and %) by mode of consent (expressed and registered opt-in and opt-out; deemed, and family consent), and numbers of families approached. The overall consent rate that included all modes of consent was 61.0% (125/205). This shows that consent rates have recovered from the dip to 45.8% in 2014/15. 22.4% (46/205) were deemed consented donors with a consent rate 60.8% (28/46), which is similar to the overall consent rate. There was a significant difference in Wales consent rates (chi-squared *p*- value = 0.009) compared to the 3 years before the introduction of the soft-opt out system. Over the same 3 year period, the consent rates in the rest of the United Kingdom also significantly increased from 58.6% (5256/8969) to 63.1% (2913/4614) (chi-squared *p*-value < 0.0001). Therefore the increase in Wales consent rates cannot solely be attributed to the change in Welsh legislation [11]. In this paper we turn to implementation processes. A summary of the final recorded consent modes and outcomes is outlined in Table 3 and Fig. 1.

**Table 3:** Summary of 18 month post implementation consent outcomes in Wales. Reproduced from [11]

Families approached by subsequent mode of consent: Deceased organ donation Wales	Total Dec 2015- May 2017 18 months
Total families approached: number of cases	205
Total cases that met the criteria for a known decision or having their consent deemed. Excludes family consent (child, not Welsh resident, lacks mental capacity)	182/205 (88.8%)
Expressed consent:	102/205 (49.7%) Registered opt in on ODR 73 Verbally expressed opt in 29
Deemed consent	46/205 (22.4%)
Family consent	23/205 (11.2%)
Total patient opt-outs:	34/205 (16.5%)
Registered opt out on ODR	8/34 (23.5%)
Verbally expressed opt out	26/34 (76.5%)
Mode of consent ascertained (consent rate)	
Total consent ascertained	125/205 (61%)
Total consent for cases that met the criteria for a known decision or having their consent deemed.	117/182 (64.2%)
Expressed consent	89/102 (87.2%)
Deemed consent	28/46 (60.8%)
Family consent	8/23 (34.7%)
Overrides by family members	
Total overrides by family members	31/205 (15.1%)
ODR overrides	12/73 (16.4%)
Other expressed overrides	1/29 (3.4%)
Deemed consent	18/46 (39.1%)

## Methods

This was an 18 month post intervention co-productive qualitative process evaluation undertaken with NHSBT, Welsh Government and multiple patient/public representatives. The process evaluation was designed using the Medical Research Council Framework [12]. The intended programme theory on which the evaluation is based is described in brief above and in detail in the protocol. The process evaluation was embedded within a before and after study to determine the impact of the soft-opt out system on consent and organ donor numbers [11].

### Aim

To explore processes that help explain initial outcomes of implementing the soft opt-out system of organ donation, and to explore SNOD practice and family experiences of SNOD intervention. The rationale being that SNODS implemented the new system and family members were involved in the decision making, so their respective behaviours created a context which impacted on the implementation of intended intervention processes and subsequent consent rates that could be explored and explained.

### Setting and participants

There are two regional NHSBT teams covering Wales; the North-West team who cover North Wales and in England the greater Manchester, Liverpool and surrounding areas, and the South Wales team. At the time of the study they had a combined workforce of 32 SNODs, 6 team managers, 2 practice development specialists, 2 regional managers and administration staff.

**Fig. 2:**
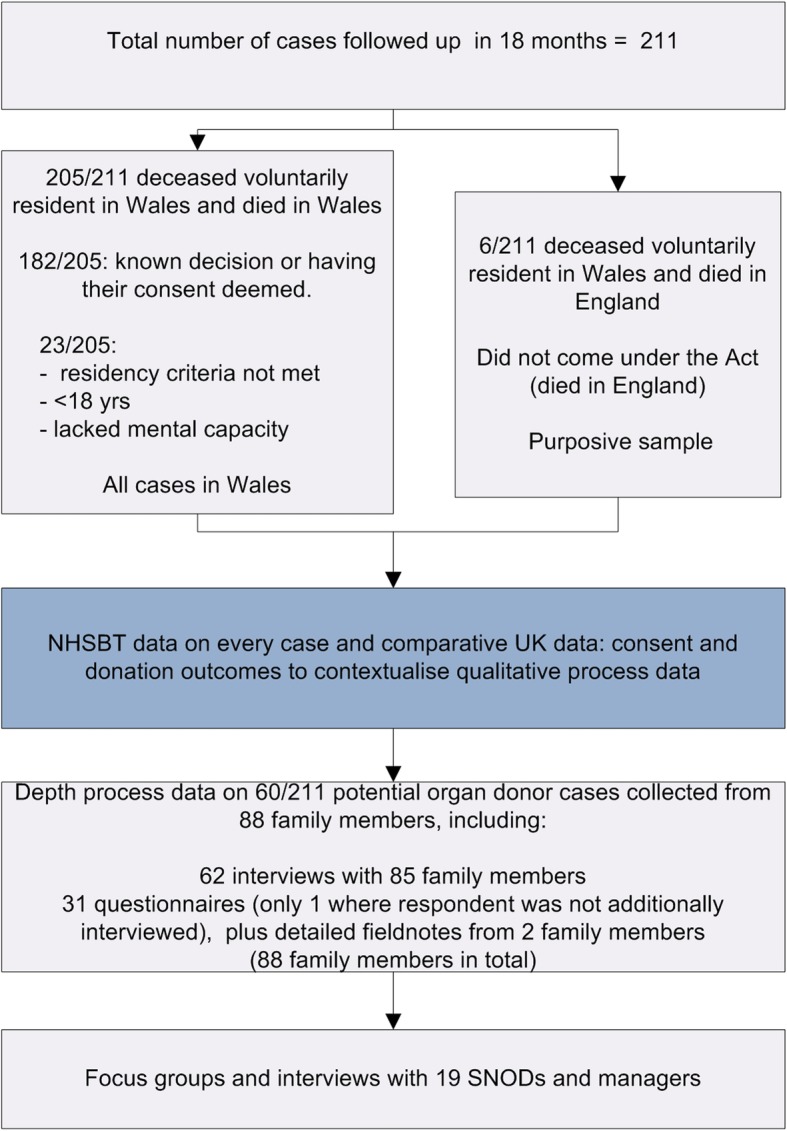
Process evaluation design and recruitment. Shows a consort-type diagram of the study design and recruitment rates. Reproduced from [6]

Data on consent pathways and outcomes were collated on all 205 potential organ donors in Wales. The relatives of all 211 potential organ donor cases were eligible to complete a questionnaire and or participate in a depth interview. This includes relatives of the 205 potential organ donor cases who died in Wales, and relatives of a purposive sample of 6 potential organ donors who lived in Wales but died at a hospital in England, making 211 cases in total. Eighty-eight family members of 60/211 cases (Table 4), and 19 professionals (15 SNODs, 2 SNOD managers, 1 regional SNOD manager and 1 SNOD practice development specialist) provided depth data on their views and experiences of implementation. The process evaluation design and recruitment are summarised in Fig. 2.

**Table 4:** Characteristics of family member participants, based on family member home postcode.

Age range (y)	*n*=	Gender	*n*=	Social deprivation scale	*n*=	Relationship to deceased~	*n*=
0–18	0	Female	53	Most deprived 5	10	Spouse or partner	40
19–35	11	Male	35	4	4	Parent or child	33
36–50	25			3	6	Sibling	6
51–70	39			2	12	Friend of long standing	9
> 71	13			Least deprived 1	30		
Total	88	Total	88	Total^#^	62	Total	88

~ Human Tissue Authority hierarchy of qualifying relationships. ^#^Not all participants provided a postcode. One deprivation score was usually calculated for each family if there was more than one participant from a single family

### Data collection

A potential organ donor was defined as a patient who is eligible for organ donation and whose family was approached for a formal organ donation discussion. Questionnaires and interview schedules were developed and refined with co-productive partners (NHSBT staff, policy makers, family members and third sector organisations. Recruitment procedures facilitated by SNODS and additional snowball sampling techniques are reported in detail in the protocol. We aimed to collect depth qualitative data from family members of a minimum number of 50 potential organ donors. Consent to contact forms were received from family members of 93 cases. Although we exceeded the original recruitment target and obtained additional depth qualitative data from family members for 60/93 cases, there was insufficient time to include the remaining 33 cases, some of whom were lost to follow up or were at a stage of their bereavement where they were still not ready to participate in an interview, but would have liked to. Recruitment was monitored to ensure that the sample represented all organ donation modes of consent and outcomes. SNODs recruited 93% of those family members interviewed and snowball sampling accounted for the remaining 7%. Family members participated in interviews with the option to complete an additional questionnaire on their views and experiences. Five interviews were conducted by phone, two in quiet public spaces (hotel and café), and the remainder at the family home. In two cases detailed fieldnotes were recorded. Interviews were conducted by female researchers who were not known to families and not involved in their clinical care. Interviews lasted up to an hour. NHSBT SNOD teams worked closely as collaborative co-productive partners to design and deliver the study. SNODs were invited to participate in interviews or one of two focus groups conducted by female researchers who had been working collaboratively with them to deliver the study in practice. Researchers also compiled detailed fieldnotes. Transcripts were not returned to participants. See Additional files 1, 2 and 3 for sample interview schedules and blank questionnaire.

### Data analysis

With consent, interviews were digitally recorded and transcribed verbatim. Free text was extracted from questionnaires. The Framework approach to coding textual data was applied [13] using NVivo Pro [14]. In this context, the programme theory and logic outlining intended behaviours, processes and outcomes were used as the Framework. Data were coded by 2 people and checked by the chief investigator. Qualitative evidence was mapped against the framework to see if and how implementation processes and outcomes were working as intended, and findings were then contextualised with quantitative observational outcomes and comparative contemporaneous data from remaining UK nations. (Table 3) [10].

### Patient and public involvement

Over 50 patient and public representatives or organisations were involved in the design, analysis and interpretation of data. A paper reporting the co-productive elements of the study is published elsewhere [6].

## Results

### Overall NHSBT perspective

SNODs initial impression was that overall implementation had gone well. This impression was supported by an increase in all modes of consent to 61.0% (125/205), showing a recovery from the dip to 45.8% in 2014/15. Examples of good practice shared by SNODs that appear to create a context to support implementation of processes as intended are summarised in Table 5. Nonetheless, consent rates in the remaining UK regions also increased over the same period, so the increase in consent rates cannot be attributed to the implementation of the soft-opt out alone.

**Table 5:** Examples of good practice that appeared to create a context to support implementation as intended

Practice	Rationale
Initial SNOD retraining and development of new procedures	All staff were retrained with new procedures. Retraining was intensive and innovative with actors and role play. A substantial budget was allocated to retraining.
Responsive implementation	Procedures were modified and SNODs and managers engaged in further retraining in response to research findings, internal audit and feedback. Managers were responsive and acted quickly. Trainers were integrated within SNOD teams.
SNOD role	Role identified by families as critical to the organ donation process. Highly valued aspects of SNOD practice included – family support, staff liaison and empathetic communication. SNODs met monthly to share practice and how they overcame implementation issues.
SNOD mindset concerning the law	SNODs transformed their thinking from having concerns about how the change would affect their conversation with family members to thinking that the new soft opt-out system provided a useful framework to structure their approach.
Research participation	SNODs actively engaged in the research process as co-productive partners.
Research co-production with patient and public representatives	SNODs and managers engaged with and were responsive to feedback from patient and public representatives.
Ongoing training	SNODs, managers and researchers engaged in an end of study training event with a crisis negotiator to directly address some of the communication issues identified by the research.

#### Overall family member perspectives on specialist nurses in organ donation practice

Family members also described the context whereby SNODs helped to successfully facilitate the consent process. We found very little evidence that the SNODs who explained the legislation to families and who challenged incorrect family perceptions of the soft-opt out had a detrimental effect on family experiences or perceptions. Family members who supported a donation decision identified the specialist nurse role as critical. Family members, almost without exception, valued the professionalism and integrity of the SNOD in the consent process. In 88 family member accounts, almost all felt the SNODs supported them through everything and improved their experience (whether they supported organ donation or not).

*‘Those nurses are some of the loveliest people you will ever meet. What they do is extraordinary. The care and compassion that they showed us at such a difficult time we will never forget it. They made us feel like something amazing was about to happen. They explained everything perfectly, helped us through everything’. (ID64 Male, spouse of deceased)*

Only one family reported that they disliked the approach made by the SNOD and did not feel comforted by the SNODs demeanour, but still went on to support organ donation.

Not all family members got to meet a SNOD as intended and had a more negative experience until they came into contact with a SNOD.

*‘We didn’t get to speak to the specialist nurses until it (organ donation) was all over. If we had I think it would have made such a difference to our negative experience. The way they came across was so lovely, not sympathetic or patronising, it is hard to explain, it was like they just understood.’ (ID08 Male, parent of deceased & ID20, Male spouse of deceased)*

#### Implementation factors

With probing, we however identified six factors that created a context which impacted on SNOD practice and negatively affected implementation, including:

- the media campaign,
- working within a more complex system,
- changing professional behaviours,
- obtaining the required standard of evidence from family members to overturn a decision,
- consent process was considered too complex, and
- additional health systems issues.

### Gaps in the media campaign

The media campaign, which is described in detail in the protocol [10], focussed on getting the citizens of Wales to share their organ donation decision with their families and friends. According to family members, the media campaign was not memorable.

*“Remember seeing the clock (counting down to the switch over) but not necessarily thinking Organ Donation. I think you need to be more explicit". This (campaign leaflet, poster) looks like a standard pedestrian information sheet. It is utterly forgettable. It just doesn’t look professional.” (ID06 Male, husband of deceased)*

At the time of airing the media campaign appeared to have most impact on getting citizens of Wales to share their organ donation decision as originally intended.

*“We heard it (media campaign) on the news in the car and I remember he was saying he thought that it was a good idea and that’s what he would definitely do and we both said the same thing –both of us would want to do it (donate their organs).” (ID05, Female wife of deceased)*

*“I was aware, definitely. Anyone in Wales who said they weren’t aware well they either didn’t/couldn’t….go to a health centre, didn’t watch television and didn’t read anything. It was a saturated campaign.” (IDS006, female daughter of deceased).*

The media campaign did not however explain the intended changed family role of supporting their relative’s organ donation decision made in life.

*“I think the campaign needs to focus more on people making decisions one way or the other and I think in order to make that decision you need people who give them more information.” (S010, female daughter of deceased)*

SNODs reported that many family members were not aware of their intended changed role and still thought that it was their decision to make. This aspect of the programme theory was complicated as the intention (family members would put their own views aside and support the donation decision made by their relative in life) was not supported by law and in reality family members still had a legal right to discount their relative’s organ donation decision). SNODs therefore faced an early barrier of having to manage families who had different expectations concerning their role than intended under the new soft-opt out system. Considerable time could be spent by SNODs in setting out the decision-making framework under the new soft-opt out. Despite SNOD explanations of how the Act was intended to work, many family members still considered that it was their decision to make as to whether their relative became an organ donor or not.

*‘We use these soft words of being the gift and when the family are sitting right in front of you and they are saying but, “if they knew that I was going to be this upset about it and that it was totally destroying me and tearing me apart he wouldn’t have wanted this so no it is not happening”, what can you do?’ (ID13 SNOD, focus group)*

Family members had varying views on their involvement in the decision-making process. Some family members wanted to be involved and to have their views on organ donation considered.

*“Families should have some say in what happens, they need to be involved in the decision”. (IDS11, female, sister of deceased)*

Whereas others felt that their decision made in life should be honoured and not overridden by family members when they died.

*“I should make my feelings clear – family has no decision to make… It should be an affirmation of who I am as a person – these are my values and this is what I want to happen (regarding organ donation)”. (ID07 female, daughter in law of deceased)*

Most SNODs acknowledged that the media campaign had helped them to support an opt-in decision made in Wales as it was likely to be a recent and informed decision as a result of the Act and the publicity campaign.

Family members who were approached about organ donation frequently lived in England and had not been exposed to the media campaign released in Wales. They were generally not aware of the differences between the opt-in system in England and the opt-out system which applied to their relative in Wales.

### Working within a more complex soft opt-out system

#### Navigating more modes of consent and establishing the correct mode of consent

Implementation of the soft opt-out system resulted in additional consent modes and geographical eligibility criteria as to which system applied (Table 2). It is common for Welsh residents to receive healthcare in England. Patients, family members and SNODs commonly had to navigate between the soft opt-out system that applied in Wales and the opt-in system that applied in England.

Under the new soft opt-out system, Welsh citizens who were over 18 years with mental capacity who died in Wales were presumed to support organ donation unless they expressed otherwise. SNODs were at times confused with implementing the presumptive approach.

*“We are supposed to be having a presumptive conversation and at the same time establishing if the deceased person had ever talked about organ donation. I don’t know if you can do both really, and it has tripped us up a bit, it doesn’t really make sense when you think about it”. (ID03 SNOD, focus group)*

SNODs also frequently found it challenging to identify the correct mode of consent in the new soft opt-out system. If the potential organ donor had not registered their decision on the ODR, SNODs needed to establish if a verbally expressed decision was made during life. This was achieved via probing questions with (often multiple) grieving family member(s). Problems arose for SNODs in trying to establish the potential donor’s verbally expressed organ donation decision as distinct from the views of the family member(s). SNODS needed to establish if there was a registered or verbally expressed decision before they could legitimately apply deemed consent.

*‘We probe the families, so their (deceased person’s) decisions and wishes are known by the rest of the family. If there is a referral to Wales and the ODR is checked and they are not on it – the communication to the team is “it might be a deemed consent”. Well it can’t be with the limited information you have at the point of referral, you have no idea until you go and speak to the family if deemed consent was ever going to be applicable.’ (ID02 SNOD, focus group)*

Many SNODs still found it difficult to unpick the deceased person’s decision made in life from the views of the (grieving) family member(s) and the Act did not help with this dilemma.

*‘You are trying to get a direct quote from the deceased person rather than a family, saying “oh I am sure he would have wanted to be a donor”, because that might be a statement that would support deemed consent or lead them to support the deemed consent but what you are trying to get at is what the patient themselves actually said not the opinion of what the family think. So if they say, “oh no he wouldn’t have wanted to do that”. Did he say that, “no – but I don’t think he would have” so if you get that sort of thing that level of evidence is not a no, it is an opinion of the family not a statement from the patient themselves.’ (ID12 SNOD, focus group)*

#### Deemed consent

Deemed consent was a new consent mode introduced in the soft-opt system. Although the media campaign worked in getting citizens of Wales to register or express their decisions, ‘doing nothing’ and having your consent deemed was poorly understood by family members. Some SNODs were unable to convince many family members that deemed consent was a positive choice that supported organ donation.

*‘When you have spoken to families where you are in a position when you have worked through the criteria, you have worked through establishing whether they discussed it[organ donation decision], and all the steps, and when you are in that clinical position with a family and consent is ‘deemable’ and then you have a reaction of, “just because they didn’t say anything doesn’t mean to say that they had no objection to it or wanted it, they would have done something about it, they just didn’t get round to it.” It is a difficult one to explain to families. Families are not sitting in a classroom learning about deemed consent. They are sat in a room having just had devastating news and it is very difficult to try and explain that (deemed consent) to them.’ (ID01 SNOD, focus group)*

Only 15/85 (17.6%) family members who were interviewed supported doing nothing as a positive choice that supported organ donation.

*‘Doing nothing causes problems for families. We should express a decision because if you do nothing and you don’t speak about it, then how is your family going to know what you want to do, they would always worry if you really wanted (or didn’t) want it.’ (ID10 Female, daughter of deceased)*

*Families are not supporting wishes either way as they didn’t know (whether relative wanted to be an organ donor or not) – then we (family) lose any element of control in what is about to happen, that’s not fair". (ID011 female, sister of deceased)*

Although there was confusion about deemed consent and what it meant, the soft opt-out system as presented in the media campaign was however seen by family members as a useful framework for decision-making.

*‘When they mentioned organ donation, I immediately thought this must be because of this new law. It helped us because none of us knew at the time what Mammy wanted.’ (ID06 Female, daughter of deceased)*

Nonetheless, due to the need to establish if a person had registered or expressed their donation decision made during life, the new consent mode of ‘deemed consent’ (whereby the person ‘did nothing’ as was assumed to have no objection to organ donation) was infrequently applied in practice.

*‘I would say a true deemed consent is actually quite rare…. You have to speak to the family and establish so many things before deemed consent applies. The law made it very clear that you did not have to go on the ODR to register a decision so we have to go to the family to establish the decision and at the point of referral we don’t know things like mental capacity…we need a lot more information before we can establish a deemed consent’ (ID06 Senior staff interview, ID07 SNOD focus group)*

*‘I am not surprised that so many potential deemed consents turn into an expressed decision. It is often my experience that families have had a conversation and some kind of yes or no decision had been made – so of course they could not be deemed.’ (ID16 SNOD, focus group)*

Mirroring the views of family members, SNODs questioned whether in some cases they might not be giving the same ‘weight’ to deemed consent as they were to the other modes of consent (such as registered or expressed consent).

*‘If you have got an opt out – registered or verbal, I would support that, it would be the same for an opt in. Where we probably fall down is do we all give the same weight to somebody who has ‘done nothing’ and I am wondering in practice if that’s the area we need more training or help with - if somebody has legitimately done nothing knowing that will make them a donor, but am I supporting that as strongly as I am supporting the opt in person.’ (ID03 SNOD, focus group)*

Some SNODs had not yet had the experience of deeming consent during the first 18 months post implementation. Their lack of exposure to this consent mode as well as recent organisational restructuring meant that many SNODs were having less frequent approach conversations and even less deemed or complex approach conversations.

*‘The frequency in which a SNOD would gain exposure from being in that particular situation of where you are able to deem consent and the family is not wanting to support it is low. I think it is very difficult to try and get your skills (communication, language, and what have you) when you have little exposure to truly practise that in practice. To have that confidence to be able to apply deemed consent and to have those very difficult conversations, and they are difficult, when we don’t get the opportunity to do that in our practice.’ (ID05 Senior staff interview, ID01 SNOD focus group)*

*‘I haven’t approached in Wales since the legislation changed so I haven’t been in that position once yet.’ (ID04 SNOD focus group)*

Several SNODs felt that their training for applying deemed consent in cases where the family might not support it, was lacking.

*‘We can sit in a class room and have education on it but to be honest it’s fine sitting there in a classroom doing it but putting that into practice when you are there alone and you have never done it and you are trying to literally kind of apply deemed consent – it is very difficult.’ (ID01 SNOD focus group)*

*‘You are looking at a very, very tiny pool of patients where we are going to be able to deem consent or have a donation conversation about it and that on a scale of being able to kind of obtain for yourself confidence and competency in doing that has to be questionable.’ (ID04 SNOD Focus group)*

*‘I think we needed more shared practice, we went from learning about it to get on with it. If you want something to work well you have got to support the people doing it. The theory is different to the practice, it really was like chalk and cheese, and the maintenance of it as well. I think Scotland and places like that they need to learn from that. I mean I know the legislation inside out, I have contributed to the training – writing scripts for actors for when the conversations don’t go as they should, and I will say you need to respond in this way. But clinical practice is a whole different world and I think we missed an opportunity on the richness of putting the theory into practice and learning from it.’ (ID02 SNOD focus group)*

Many SNODs expressed further concerns that ‘pushing’ a deemed consent could do more harm to the public perception of organ donation, and to the relationships that NHSBT have built up with the NHS Units and clinical staff.

*‘I’m also quite mindful of the damage we can create if we probe and probe, push the family into deemed consent – if they are clearly against donation going ahead the potential damage that one can make for future donations in Wales, and, aside from all that, if we have a medical team that doesn’t agree with the decision, if we probe, if we push that family into organ donation how would we know if they (clinical staff) are going to follow through with it…. Consultants will also be quite deterministic about their patient and rightly so.’ (ID03 SNOD focus group)*

#### Balancing the dual roles of supporting the decision of the deceased person and supporting the bereaved family

The new soft opt-out system and associated retraining of SNODs was designed to help the family to support their relative’s donation decision made in life, whilst acknowledging that family members could still override their relative’s decision. At the same time SNODS were supporting the family through the initial bereavement and trauma. This dual role was not straightforward for SNODS to implement in practice. Some SNODs found it difficult to get the balance right between caring for the family and focussing on supporting the organ donation decision of the deceased person.

*‘I can be quite paternalistic and quite conscious and probably from my past of bereavement care in making sure that we are not making things worse for people and I don’t ever want to be in a position to make people (family members) feel uncomfortable about whichever decision they are making…. I don’t think that me as a person necessarily can influence and change somebody’s mind and it doesn’t matter what the government tell me and what they instruct should be there as evidence, at the end of the day I am a human, open, honest person who hopefully is caring for another family and if they don’t want something to go ahead for whatever reasons they have to live with that. If they are overturning somebody’s wish – I wouldn’t say I would walk away from it, I would do my best, but I will perhaps not go to the lengths that somebody else would and that’s my belief system.’ (ID01 SNOD, focus group)*

### Changing professional behaviours

Many SNODs indicated that the initial implementation of the Act did not have any direct influence on the way they practiced.

*‘We don’t act any differently when we step over the border (between England and Wales)….. Whilst being aware of the law we would all act in what we felt was in their (family member’s) best interests.’ (ID02 SNOD focus group)*

Not all SNODs were able to adopt the required changes to the language that they used with family members from establishing the ‘wishes’ of the deceased person in the opt-in system to establishing their ‘donation decision’ in the soft opt-out system. SNODs continued to mirror the language used by families, which generally focussed on ‘wishes’ and not ‘decisions’. Many of the quotes reported here still use the language of ‘wishes’ mixed in with ‘decisions’. Two thirds of the SNODs worked in England and Wales and had to accommodate both opt-in and out-out systems at the same time so it is easy to see how a legacy of language spilled over into the new soft opt-out system in Wales. In England, if a person has not opted in (via registration or verbally) the family are legally able to make the decision on what they want not necessarily (but hopefully) with regard to the wishes of the deceased.

As reported above, the media campaign did not focus on the changed role of families in supporting their relative’s organ donation ‘decision’. Families therefore continued to see organ donation as what their relative might have wished for (or not), rather than a firm decision made during their lifetime.

*‘We do loads of work around language and consent. One of the key things that we feel is that we use soft words like ‘gift’ which sits along ways like wishes and that’s difficult because deemed consent in itself is a very technical language and we are talking about softening it and approaching these families and using words like gifts and all these things. That is also the complexity the two different languages I think.’ (ID13 SNOD focus group)*

Some SNODs did however feel that they had probably changed their practice when implementing the soft opt-out system in Wales.

*‘For me certainly an opt-in, it gives me complete confidence to support a family that I am approaching probably more so (in Wales) than in England cause the opt in is often – it’s very informed it is on the basis of the law changing a lot of the time…… this gives me the confidence to maybe to use different language when I speak to families about the way we kind of choose to make organ donation decisions in Wales so it would probably go some way to change my practice.’ (ID03 SNOD focus group)*

SNODs were also required to ascertain the last known decision of the deceased person, which may have changed a previous registered or expressed decision. The language of ‘wishes’ and ‘decisions’ was also used in ascertaining the last known decision.

‘I think and that is the difficult thing because we have gone in talking about have they had a known wish and it’s kind of lost in terms of approaching for a presumptive wish which is deemed consent and that’s been the trickiness of the language (in the Act) I think, and I am not sure we are completely there 100% yet.’ (ID15 SNOD focus group)

### Obtaining the required standard of evidence from family members to overturn a decision

Irrespective of the Act, the media campaign, and SNOD retraining, SNODs found it challenging to prevent family member(s) overriding an organ donation decision regardless of how the decision was made.

*‘Maybe they (family members) think it is their exertion of their control. They might feel that it has been taken away when the law changed. It was a naïve assumption as people didn’t know what it was going to actually involve. Maybe for some (overrides) it’s the recognition and the acknowledgement that the law has changed but ultimately they want the say over their relative in that clinical setting, at that time, that it will be what they want for them or what they don’t want for them rather than what some nurse, doctor or government is going to tell them whilst they are in hospital.’ (ID16 SNOD, focus group)*

The Act outlines clear standards of evidence (written or witnessed conversation) for overturning any deceased person’s decision made in life, including deemed consent. These standards were unrealistic for SNODs to implement in practice. As shown in Table 3 there were 31/205 (15.1%) family member overrides of organ donation decisions made by their relative in life. 12/73 (16.4%) ODR, 1/29 (3.4%) were expressed decisions and 18/46 (39.1%) deemed consent overrides. None of these overrides was undertaken with the required standard of evidence. There were no cases where an opt-out decision was overridden.

*‘We know the language of the Act was very specific about standards of evidence, but that was very unrealistic. I think that was an ideal rather than reality. The reality of having something written that they have kind of overturned or what have you I think in practice it is probably very unlikely that we would have that to assist us.’ (ID18 SNOD, focus group)*

*‘The language they (the family) were using was that, "yeh we know that is the legislation but it is not going to happen’. (ID15 SNOD, focus group)*

SNODs illustrated many cases where they felt that they were powerless to challenge the family.

*‘The other day I had an ‘opt-in’ case. When I went in for consent the family got really upset and said, “he wouldn’t want to be like this in intensive care he wouldn’t want to be the way he is in agony for 24 hours”, and then we offer to show them the organ registration form. They totally point blank refused and wouldn’t even look at it and said “actually I think it was me who filled the form in for him and I ticked the box for him”. Now we can’t prove that in our enquiries.’ (ID15 SNOD, focus group)*

#### Concern that the last known decision was sometimes being used to override a previous opt-in decision

SNODs expressed concern that individual family members may be using the ‘last known decision’ to override a previous opt-in decision. A last known expressed opt-out decision frequently appeared to be contrary to what all the other family members were saying about their relative wanting to be an organ donor. Recent reversals of previously registered or expressed ‘decisions’ were not recorded as ‘overrides’.

*‘If you question the family member though, they could say oh well we had a conversation and I promised I would do that (opt them out on the ODR) this week – how are you ever going to argue against that – there always could be some plausible excuse but I agree it doesn’t sit comfortably with you does it when you have found that has happened.’ (ID08 SNOD, focus group)*

*‘The family had said there were recent communications with their mum and she’d said she wouldn’t want it (organ donation) to happen yet she had put herself on the ODR about 10 years previously’. (ID08 SNOD, focus group)*

Family members also expressed surprise and concern when a recent conversation about organ donation was said to have happened with the deceased person.

*‘Well we were all just so surprised when she (daughter) said that she had talked about it with mam last week and that she expressed that she didn’t want to be a donor. It was at odds with what I knew and what I had always presumed - that she wanted to be an organ donor…… In the end we went with what my daughter wanted.’ (ID57 Male, spouse of deceased)*

Based on their experiences of feeling powerless and not able to pursue the required standard of evidence to override a decision, many SNODs felt that the Act had not gone far enough in supporting them in their practice.

*‘When people knew that the law was changing and when the education and the awareness campaign was put into place people did start talking about it so that is as valid as registering your decision or doing nothing isn’t it and families were encouraged to talk. (ID02) It is a shame really that they (citizens of Wales) didn’t have a mandated choice it would have been a lot simpler wouldn’t it.’ (ID03 SNOD, focus group)*

SNODs were also realistic about the need to keep families involved in the organ donation process and the unlikelihood of the law changing to a hard opt-out that did not consult with families.

*‘The truth is the Act will only be effective kind of when the people or the SNODs lay down the letter of the law. But I don’t see any government that would be able to put a law in place that could deem consent without the support of the family. We need the family to come on this journey for health questions etc.’ (ID18 SNOD, focus group)*

SNODs recognised that more people would need to support organ donation and overrides would need to be reduced in order to meet the organ donation target of 80% consent rate for 2020 set by Welsh Government. SNODs suggested more work to educate children might support them in reaching the consent targets.

*‘All the changes that have come in we are still not quite reaching where we want to be we still need to keep evolving and trying to address this situation as well. The education team is trying to do that and we have just got to keep plugging away. I think is the answer and whether it needs to be a societal change which I suspect it does which we have already alluded to is always our kids. I think that is essentially where we need to start and it seems to be it needs an approach from all ages whether it would be the education service, health service, government it somehow it has got to get into the consciousness of the British people.’ (ID06 Senior staff interview, ID09 SNOD focus group)*

### Overly complex consent process

SNODs were required to go through a lengthy form to get family agreement for each of the organs to be donated. In some cases family members reported that there was too much over qualifying after they had supported their relative’s decision to donate.

*‘Honestly I found the whole thing very irritating. The care and their professionalism was outstanding I cannot fault that – everybody there was superb, brilliant. But it just went on and on, asking about this organ and that organ, and was I sure and was I absolutely sure. It wasn’t my decision in the first place but I supported it, why couldn’t they just get on with it. My suggestion is some sort of branching system for those who yes to everything it just goes ahead and then for those who want more details there is a system for them as well.’ (IDAS006, Male, spouse of deceased)*

### Other health systems issues

Health systems issues that were unrelated to the soft opt-out system also affected and prevented family member support for an opt-in donation decision. Of 31 families who overrode an opt-in decision*:

- The time frame to organ donation was considered too long (8/31).

*“At the start we thought yes of course, but it all went on too long. In the end we all thought this isn’t dignified. We don’t regret saying no, we held on as long as we could.” (ID013 Female, daughter of deceased)*

- No Specialist Nurse in Organ Donation was available (3/31 family members who overrode an opt-in decision and 19 (186/205) cases overall where a SNOD was not present).
- The perceived (poor) quality of general NHS care (3/31).

*“In the back of my mind is the poor treatment that she had and the thoughts keep coming through did she have the best care? ….. I lost all my trust with the doctors in the hospital because they treated her absolutely diabolical.” (ID012 husband of deceased)*

* More than one reason could apply.

In addition, family members said that it was not always possible for them to stay at or near the hospital at no charge whilst donation proceeded and so they were not able to support their relative’s decision to donate. Few families had access to ongoing bereavement care, especially if they lived far from the hospital where their relative died.

The purposive sample of families of 6/38 cases who lived in Wales and died in England and were managed under the opt-in system in England were confused when Welsh patients died in English hospitals as to which system applied.

## Discussion

SNODs generally did not think that they were having difficulties in implementing the Act as intended (which is similar to findings from interviews conducted by Welsh Government) [15]. Nonetheless, *tricky, challenging* and *difficult* were the most frequently used terms by SNODs when it came to describing specific aspects of implementation. The main weakness in the media campaign was that it did not spell out the intended changed role of family members to one of supporting their relative’s donation decision made in life. Although SNODs tried hard to mitigate this communication deficit, families frequently did not alter their misconception that it was their decision to make. The initial programme theory of how the intervention was intended to work was overly optimistic that relatives would consistently put their own views aside to support the donation decision of their relative made in life. In overriding an organ donation decision, relatives are acting within the law, even though their action is not in the spirit of honouring the donation decision of the deceased person made in life.

SNODs appeared to be interpreting the Act and its implementation to suit their particular situation and were doing what they saw as best for the family member(s). This included the use of language, using softer terms like ‘wishes’ and not requesting from family members the required standard of evidence to override a donation decision made by a deceased person during life. This was not understood to be an implementation issue by SNODs who felt that they were doing what was best for the family by softening terms so the family felt comforted during traumatic events. Worries about clinical repercussions, family upset, and longer-term damage to organ donation consent rates also prevented SNODs from trying to implement the required standard of evidence to override a donation decision as outlined in the Act. Arguably these standards are impracticable. To ask grieving family members to produce written evidence or a witness to corroborate a change of organ donation decision was felt to counteract the necessary care and support SNODs provided to grieving families. This also illustrates the dilemma as to whether the role of the SNOD is primarily to help honour the decision of the person made in life or to support and comfort bereaved family members when their relative is a potential organ donor. The ‘enforcer’ role attributed to SNODs did not however appear to have a detrimental effect on family members.

The Act gave decisions about organ donation to citizens of Wales to make during life and created a more complex system with additional consent modes. It did not sufficiently address the complexities of establishing these decisions through bereaved family member(s) and did not provide SNODs with realistic tools to mitigate family members overriding their relative’s organ donation decision made in life.

‘Doing nothing’ and having your consent deemed was complicated for family member(s) to understand and key messages in the media campaign were not fully understood. This made it difficult for SNODs to argue the case for ‘doing nothing’ as an informed choice which supported organ donation. Nonetheless, the consent rate for deemed consent (60.8%) was the same as the overall consent rate (61%). There is however no way of telling if these families would have also consented under the former opt-in system. It was also particularly challenging for SNODs to unpick the deceased person’s donation decision when not registered on the organ donor register and there was a contradiction in how this should be addressed in initial implementation. A presumptive approach could not happen until the SNOD had established that the deceased person ‘did nothing’, but this conversation happened through the family member(s). During these conversations the family had opportunities to assert their own views, feelings, worries and concerns and in 31 cases stop the donation proceeding by overriding the deceased person’s decision made in life.

We could not locate other similar nurse-led implementation studies with which to compare findings. Quigley *at al.* [16] provide a retrospective analysis of theoretical and pragmatic considerations that appear to positively influence the success of the medically-led opt-out system in Spain. They present no analysis as to whether the key principles were implemented as intended and only briefly mention nurses in relation to their collaborating role with the transplant coordination network. Likewise, Kwek et al. [17] report their medical perspectives as to why introduction of a soft-opt out system implemented 21 years previously in Singapore had no impact on donor numbers. They called for further reorganisation (for example to identify potential donors earlier), and did not mention nurses as playing a significant role in what appeared to be a medically led system.

### Strengths and limitations

This was a large co-productive study in partnership with NHSBT and multiple key stakeholders. Many of the findings and subsequent recommendations have already been implemented and/or are being addressed in Wales. A strength was that data were collected on all 205 organ donation cases in Wales, and the process evaluation was large involving 107 participants, and conducted by independent researchers. Family members preferred to be interviewed face to face which yielded richer data, rather than opting for completing a questionnaire. We were not able to include other members of the NHS workforce in data collection. Nonetheless, many NHS staff (including intensive care consultants) and several co-authors who were NHS employees served as co-productive partners.

### Implications for policy, practice and research

A media campaign focussing on the changed role of the family needs to be implemented prior to changing to a soft opt-out system. In Wales a new post implementation campaign was commissioned based on the findings from this study.

Pre-implementation SNOD training appeared to be too focussed on deeming consent and taking a ‘presumptive approach’. Some rethinking of processes and ongoing training was required to establish the deceased person’s decisions as separate from the views of the grieving family members.

The low frequency of approaches (especially for deemed consent) meant that some SNODs were not gaining sufficient practical experience to further develop their skills. NHSBT had already recognised this gap in learning and implemented a new ‘Specialist Requester’ role in England prior to the legislative change, which will subsequently be implemented in Wales. Implementation of Specialist Requesters was primarily undertaken to increase exposure and experience in ascertaining consent regardless of mode of consent and any legislative change. The Specialist Requester (ideally a former high performing SNOD in obtaining consent who undergoes further retraining) focuses on approaching the family and consent, whilst the SNOD role focusses on subsequent family support.

It remains uncertain whether the final organ donation decisions were genuinely the decisions of the deceased person as expressed in life, what the family member(s) honestly thought or felt the deceased person would or would not want, or if the family member(s) were putting their own views and opinions across in place of the deceased person’s views. The required standard of evidence to override a donation decision needs revisiting and additional practical solutions are required that are easier for Specialist Requesters and SNODs to implement. These important concerns and issues need to be further explored in future longitudinal research. The roles of nurses need to be better described in studies concerning organ donation so that international comparisons can be made. The TiDieR Framework for describing and replicating interventions could be used to increase the transparency of nursing [18]. Finally, the impact of the legislation on the wider multi-disciplinary team needs to be explored.

## Conclusion

This is the first detailed process evaluation of specialist nurse implementation of a soft opt-out system of organ donation. Scotland, England and the Netherlands are in the process of introducing soft opt-out systems. There is an opportunity to learn from initial implementation in Wales, by acknowledging good practice, and implementation gaps and opportunities to further improve processes and practices. The key message is that despite retraining, SNOD practice did not radically change overnight to accommodate the new soft opt-out system. Policy makers and health service managers should not assume that SNODs simply need more time to implement the soft opt-out as intended. Additional modification of processes and further training and support is required to help SNODs with ongoing implementation as originally intended. The media campaign supporting implementation should explain the intended role of the family in the decision-making process.

## Additional files

Additional file 1: Professional focus group interview schedule. Contains an outline of the focus group format and questions asked. (DOCX 32 kb)

Additional file 2: Family member interview protocol. Contains an outline of the interview format and questions asked. (DOCX 78 kb)

Additional file 3: Family member questionnaire. Contains the questionnaire given to family members. (PDF 49 kb)

## Data Availability

Qualitative dataset is not available because participants did not consent to this as part of our study recruitment process. In addition since this is a qualitative study, involving participants from one locality, there is a possibility that material in the transcripts could identify participants.

## Abbreviations

NHS: National Health Service
NHSBT: National Health Service Blood and Transplant
ODR: Organ Donor Register
RINTAG: Research, Innovation and Technology Advisory Group
SNOD: Specialist Nurse in Organ Donation
UK: United Kingdom
