# Supplementary material for: Process evaluation of specialist nurse implementation of a soft opt-out organ donation system in Wales
Source: BMC Health Serv Res. 2019 Jun 24;19:414. doi: 10.1186/s12913-019-4266-z (PMC6591913; doi:10.1186/s12913-019-4266-z)
Supplement: Supplementary file 2 — Family member interview protocol. Contains an outline of the interview format and questions asked. (DOCX 78 kb) [file 12913_2019_4266_MOESM2_ESM.docx]

**Focus Groups – SNODS**

**Check that:**

**1. Consent forms are filled out correctly and completed.**

**2. Opportunity for questions is provided.**

**3. Anonymity of participation is clear.**

**4. Recorders are on.**

**5. Brief explanation of project, where we are in the data analysis and why we are asking for SNOD input is understood. *e.g. ‘The impact on practices and changes has been explored by the Welsh Assembly Government funded qualitative study conducted by Beaufort Research.***

***We have limited time today so we will be focussing and asking more about specific issues to help with our interpretation and understanding especially in areas where we have less data.***

***Your confidential insights are vital to understanding any impact of the changes and identifying any areas for improvement.***

**Begin focus group:**

**1.One area we need more clarity on is the language of deemed consent, known wishes and expressed decisions and what is happening in terms of decision making and outcomes.**

- The language of the Act is about expressed decisions, much of the language now is about known wishes, is this deliberate?
- What qualifies for you as an ‘expressed wish/decision’? Check for agreement with the SNODS. Is this different than originally envisaged?
- We have seen a high number (share figure) deemed consents transform into expressed decisions – opt out, can you tell us more about this with specific examples.
- Do you have any views on the appropriateness of when the ‘known wish’ changes the deemed consent? (share example)
- What strategies do you have to manage these cases?
- There have been cases where family members are opting their loved ones out on the ODR when they are in critical care – why do they feel they need to do this?

**2. A second area that we need more clarity on is ODR and Deemed overrides**

- The act is presumptive. What is happening so that ODR and Deemed overrides are commonly occurring? Please give specific examples
- How do you feel when this happens, ad what else do you need to make it less frequent?
- How do you capture an ODR/deemed override?
- Has anybody declared a known wish to override an Opt Out – is this possible?

**3. Miscellaneous**

*Consent rates have increased and it appears that families are having more conversations about organ donation.*

What are your general views on what has/has not worked since implementation of the act?

What has been most helpful in terms of implementing the act and changes in your practice

What has been most unhelpful – in terms of implementing the act and changes in your practice

Anything else you would like to mention that you feel would help us with our understanding and analysis.

Have the views of families changed since the act was implemented: do they view organ donation as a gift or a sacrifice?

**Thanks and close.**

Summary of factors influencing supporting/not supporting Organ Donation Decision from SNODs

|  | In ODR | Out ODR | In expressed | Out expressed | Deemed |
| --- | --- | --- | --- | --- | --- |
| Supported (Reasons why) |  |  |  |  |  |
| Not supported  (reasons why) |  |  |  |  |  |
