# Supplementary material for: Process evaluation of specialist nurse implementation of a soft opt-out organ donation system in Wales
Source: BMC Health Serv Res. 2019 Jun 24;19:414. doi: 10.1186/s12913-019-4266-z (PMC6591913; doi:10.1186/s12913-019-4266-z)
Supplement: Supplementary file 3 — Family member questionnaire. Contains the questionnaire given to family members. (PDF 49 kb) [file 12913_2019_4266_MOESM3_ESM.pdf]

FORM C: Organ Donation: Questionnaire for Family/ Close Friends/ Appointed Representatives

Thank you for agreeing to fill out this questionnaire. Anybody involved in the donation decision can choose to fill out a questionnaire. The questions are quite short but feel free to add more details at the bottom. There is an option to complete this online if you prefer: https://bangor.onlinesurveys.ac.uk/organ-donation-project-questionnaire. There is no limit to the number of questionnaires per family. By returning the completed questionnaire to the research team at Bangor University, it will be assumed that you have given your consent for the researchers to analyse and use the data.

1. Are you: spouse or partner ☐ parent or child ☐ brother or sister ☐ grandparent or grandchild ☐ niece or nephew ☐ stepfather or stepmother ☐ half-brother or half-sister ☐ friend of long standing ☐ Appointed Representative ☐

(*An appointed representative is a person appointed by your loved one or close friend during their lifetime to convey their organ donation decision after their death.)*

2. Did your loved one or close friend pass away in a hospital in:
Wales ☐ (go to Q.4) England ☐ (go to Q.3)

3. Did you know that the changes to consenting to organ donation in Wales did not apply in England? Yes ☐ No ☐ Uncertain ☐

4. On the organ donor register, did your loved one or close friend:
Opt In ☐ Opt Out ☐ Appoint a Representative ☐ Do Nothing ☐

5. Did you know about your loved one or close friend's decision on the organ donor register?
Yes ☐ No ☐ Uncertain ☐ Non Applicable ☐

6. Did your loved one or close friend ever discuss their donation decision with you?
Yes ☐ No ☐ Uncertain ☐ Non Applicable ☐

7. Did you support your loved one or close friend's organ donation decision after they passed away?
Yes ☐ No ☐ Uncertain ☐ Non Applicable ☐

8. Did you feel able to support the deemed consent of your loved one or close friend when the changes to consenting to organ donation in Wales were explained by a specialist nurse in organ donation?
Yes ☐ No ☐ Uncertain ☐ Non Applicable ☐

9. Do you consider yourself the decision maker on behalf of your loved one or friend who passed away? Yes ☐ No ☐ (go toQ.11)

10. As the decision maker, did the changes to consenting to organ donation in Wales help you at this difficult time?
Yes ☐ No ☐ Uncertain ☐

Please turn over the page now

11. Was there any form of disagreement between anybody involved in the donation discussion?
Yes ☐ No ☐ Uncertain ☐ Non Applicable ☐
If yes, did the changes to consenting to organ donation in Wales help to reach agreement?
Yes ☐ No ☐ Uncertain ☐ Non Applicable ☐

12. When somebody sadly passes away and they meet very specific criteria in Wales, if they have NOT registered a decision on the organ donor register or have NOT discussed their organ donation decision with family and friends, it means that their consent to organ donation can be deemed.

On a scale of 1 to 10 (1 = not at all and 10 = fully understood)

How well would you say you understood the changes to consenting to organ donation in Wales BEFORE you spoke to a specialist nurse in organ donation?
1 ☐ 2 ☐ 3 ☐ 4 ☐ 5 ☐ 6 ☐ 7 ☐ 8 ☐ 9 ☐ 10 ☐

How well would you say you understood the changes to consenting to organ donation in Wales AFTER you spoke to a specialist nurse in organ donation?
1 ☐ 2 ☐ 3 ☐ 4 ☐ 5 ☐ 6 ☐ 7 ☐ 8 ☐ 9 ☐ 10 ☐

13. Had you heard or read about the changes to consenting to organ donation in Wales from the media campaign (Radio, TV, newspapers, postal leaflet etc.)?
Yes ☐ No ☐ Uncertain ☐

14. Have the changes to consenting to organ donation encouraged you to register your decision on the organ donation register?
Yes ☐ No ☐ Uncertain ☐ Non Applicable ☐

Other comments: Please tell us anything else that you think is important.

____

____

____

____

____

____

____

____

____

____

____

____

____

Thank you kindly for your responses at this difficult time. If you would like to share your stories with us in more detail, please fill out Form D (consent to be contacted for interview) and return with this questionnaire in the pre-paid envelope. We look forward to hearing from you.
